# Supplementary material for: Dominance of S. cerevisiae Commercial Starter Strains during Greco di Tufo and Aglianico Wine Fermentations and Evaluation of Oenological Performances of Some Indigenous/Residential Strains
Source: Foods. 2020 Oct 26;9(11):1549. doi: 10.3390/foods9111549 (PMC7692326; doi:10.3390/foods9111549)
Supplement: Supplementary file 1 [file foods-09-01549-s001.pdf]

Supplementary Materials:

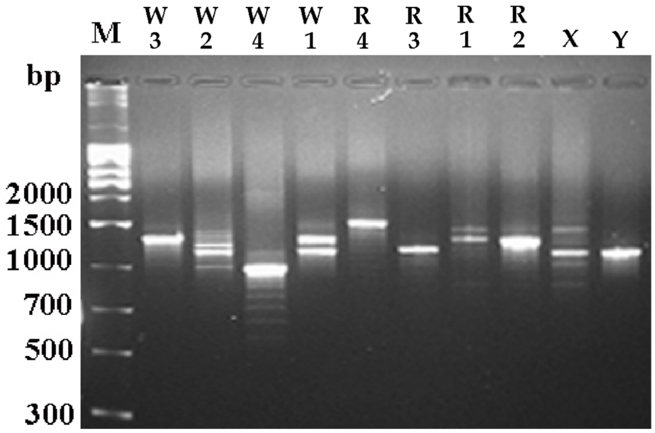

Figure S1. Minisatellite *DAN4* patterns of commercial starter strains used in this study.

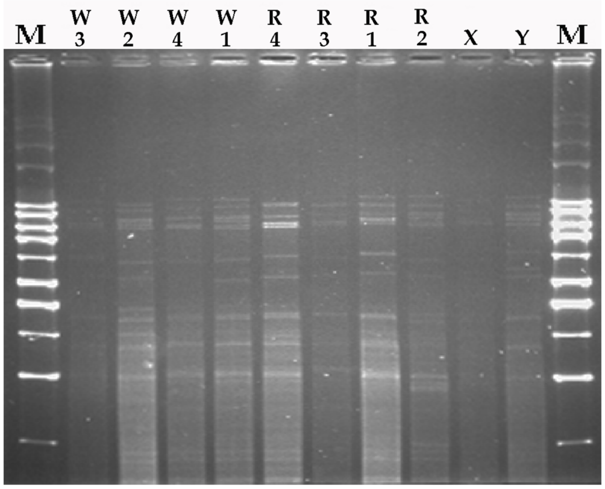

Figure S2. mtDNA-RFLP (*Rsa*I) patterns of commercial starter strains used is this study.
